# Comprehensive pulmonary rehabilitation for post-tuberculosis lung disease: a narrative review

**DOI:** 10.3389/fmed.2026.1899760

**Published:** 2026-09-25

**Authors:** Rui Li, Kai Ling Chin, Qiuyi Li, Yaxi Zhao, Yang Yang, Luis Anibarro, Ana Chouza Piñeiro, Maria E. Sarmiento, Armando Acosta

**Affiliations:** 1 Department of Rehabilitation Medicine, The Second People’s Hospital of Kunming, Rehabilitation Hospital Affiliated to Kunming University, Kunming, China; 2 Department of Biomedical Sciences, Faculty of Medicine and Health Sciences, Universiti Malaysia Sabah, Kota Kinabalu, Malaysia; 3 Borneo Medical and Health Research Centre, Faculty of Medicine and Health Sciences, Universiti Malaysia Sabah, Kota Kinabalu, Malaysia; 4 Department of Clinical Nutrition, The Second People’s Hospital of Kunming, Rehabilitation Hospital Affiliated to Kunming University, Kunming, China; 5 Department of Geriatrics, The Mental Hospital of Yunnan Province, The Affiliated Mental Hospital of Kunming Medical University, Kunming, China; 6 Tuberculosis Unit, Infectious Diseases, and Internal Medicine Department, Complexo Hospitalario Universitario de Pontevedra, Pontevedra, Spain; 7 Immunology Research Group, Galicia Sur Health Research Institute (IIS-GS), Vigo, Spain; 8 Independent Researcher, Valencian Community, Spain

**Keywords:** multidisciplinary approach, outcome measures, post-tuberculosis, pulmonary rehabilitation, quality of life, telemedicine

## Abstract

Around 155 million people worldwide are tuberculosis (TB) survivors, many of whom experience post-tuberculosis lung disease (PTLD), characterized by structural lung abnormalities, impaired pulmonary function, persistent respiratory symptoms, reduced exercise capacity, and poorer quality of life. The burden of PTLD is particularly important in low- and middle-income countries (LMICs), where access to pulmonary rehabilitation (PR) and long-term post-TB care remains limited. This narrative review synthesizes current evidence on comprehensive PR for PTLD and considers its implementation across diverse healthcare settings. Available studies suggest that PR can improve exercise capacity, respiratory symptoms, functional status, and health-related quality of life in individuals with post-TB pulmonary impairment, although PTLD-specific evidence remains limited and heterogeneous. Comprehensive rehabilitation extends beyond exercise training to include breathing and airway-clearance strategies, nutritional support, psychological and psychosocial care, patient education, and tele-rehabilitation. Where direct PTLD-specific evidence is limited, established evidence and principles from rehabilitation in other chronic respiratory diseases may inform the adaptation of these components to post-TB care. Key implementation challenges in resource-constrained settings include limited rehabilitation infrastructure, workforce and financial constraints, geographical barriers, and poor integration of rehabilitation into routine TB services. Context-adapted approaches, including simplified, community-based, home-based, and digitally supported models, may improve accessibility and feasibility. Integrating multidisciplinary rehabilitation into post-TB care may help address the long-term physical, psychological, and social consequences of TB; however, further PTLD-specific trials and implementation research are needed to establish optimal programme components, delivery models, and long-term effectiveness.

## 1 Introduction

Tuberculosis (TB), caused by *Mycobacterium tuberculosis* (Mtb), is an airborne disease that primarily affects the lungs. Globally, an estimated 10.8 million people fell ill with TB, with a total of 1.25 million deaths in 2023 (1). About 400,000 cases were classified as multidrug-resistant (resistance to both rifampicin and isoniazid)/ rifampicin-resistant TB (MDR/RR-TB) (1). The emergence and transmission of drug-resistant tuberculosis (DR-TB) are linked to multiple factors, including poor treatment adherence, incomplete or suboptimal treatment regimens prescribed by healthcare providers, failure to diagnose MDR strains, and inadequate healthcare systems that lack the resources to implement comprehensive DR-TB control programs (2).

In 2020, an estimated 155 million post-TB survivors, or 1 in 50 people globally, were reported (3). Despite a surge in research interest and recently published clinical standards for managing post-TB lung disease (PTLD), this condition remains largely neglected at a programmatic level, especially in high TB burden countries. Traditional TB treatment focuses on standardized microbiological outcomes, often overlooking the overall health and well-being of the patient. As a result, many programs discharge patients after treatment completion, regardless of residual lung damage, which is severe in 10%–15% of survivors, further exacerbating the risk of long-term morbidity (4). This underscores the importance of pulmonary rehabilitation (PR) as a key intervention, not only to alleviate respiratory morbidity in post-TB survivors but also to support their long-term recovery, enhance lung function, and improve overall quality of life as part of comprehensive care.

### 1.1 TB pathophysiology and lung damage

Mtb enters the body through the inhalation of aerosolized droplets produced by actions such as coughing, sneezing, or singing. Once in the lung, the innate immune response is activated, and the bacteria are engulfed by alveolar macrophages. However, Mtb has evolved several survival strategies within the phagosomes such as inhibits the maturation and acidification of phagolysosomes, suppresses oxidative stress, and prevents apoptosis and autophagy (5). By halting these processes, Mtb creates a favorable environment within the macrophages that allows it to evade the immune system, thereby enhancing its survival. During the early stages of infection, as the bacteria proliferate within the macrophages, the immune system responds by formation of a granuloma, a structured immune cell cluster that attempts to contain the infection. Within granulomas, Mtb may remain dormant and asymptomatic, but it may reactivate if immunity weakens (6).

About 5%–10% of Mtb-infected individuals will develop active TB either as primary TB (common in children, malnutrition or immunocompromised individuals) or secondary TB (reactivation due to immune distruption) (7). In active TB, the granulomas break down, and the bacteria can spread through the lungs causing pulmonary TB (PTB). Lung damage and inflammation occur due to the ongoing immune response and bacterial proliferation. Even after completing TB drug treatment, many individuals face chronic respiratory symptoms such as a persistent cough, shortness of breath, chest pain, and fatigue. These symptoms are often the consequence of structural lung abnormalities, which include bronchiectasis (damage to the airways causing them to widen and become scarred), bronchial stenosis (narrowing of the bronchial tubes), cavitation (formation of hollow spaces in lung tissue), fibro-nodular scarring, and pleural thickening. These structural changes alter lung compliance (ability to stretch and expand), gas exchange (the efficiency of oxygen and carbon dioxide exchange), functional lung volumes, and airflow. As a result, lung function abnormalities such as restrictive, obstructive, and mixed defects can develop, with each episode of PTB increasing the risk of irreversible respiratory impairment (8). Beyond lung disease, they also face risks of cardiac complications (e.g., pericardial and myocardial disease, valvular disease, and rhythm disturbances), neurological complications (e.g., TB meningitis and spinal TB), and psychiatric illnesses, further contributing to the long-term health challenges faced by these individuals (8).

### 1.2 Anti-tuberculous drugs treatment

Treatment for TB is a lengthy process, primarily involving the use of first-line anti-TB drugs. The most common treatment for drug-susceptible TB is a 6-month regimen of rifampin, isoniazid, pyrazinamide, and ethambutol (6-RIPE). In a case of smear-negative, culture-negative, noncavitary pulmonary TB, where the clinician determines the disease is likely paucibacillary or has a low mycobacterial burden, a 4-month regimen of rifapentine and moxifloxacin is recommended for individuals over 12 years old, weighing more than 40 kilograms, or for those with HIV, provided their CD4 count is at or above 100 cells/μL (9). The efficacy of this treatment is non-inferior to the standard 6-RIPE (10).

The treatment for DR-TB is a longer process, frequently lasting 18 to 24 months or even longer, depending on the specific drug resistance patterns. However, in 2022, the World Health Organization (WHO) recommended a shorter 6-month regimen consisting of bedaquiline, pretomanid, and linezolid, with or without moxifloxacin (BPaL and BPaLM, respectively), replacing the previous 9- to 18-month regimens used for MDR/RR-TB treatment (11–13). Despite this recommendation, a study on the accessibility of BPaL(M) regimen drugs found that only about 52.3% of countries in the Europe region had access to them (14). If the 6-month MDR/RR-TB regimen is unavailable, a 9-month oral regimen includes 6 months of bedaquiline with 4 months of levofloxacin/moxifloxacin, ethionamide, ethambutol, high-dose isoniazid, pyrazinamide, and clofazimine (extendable to 6 months if sputum smear positive after 4 months). This is followed by 5 months of levofloxacin/moxifloxacin, clofazimine, ethambutol, and pyrazinamide. Ethionamide can be replaced with 2 months of linezolid (600 mg daily) (15). In 2024, the WHO recommended two new oral regimens for MDR/RR-TB: a 6-month regimen consisting of bedaquiline, delamanid, linezolid (600 mg), levofloxacin, and clofazimine (BDLLfxC), and 9-month regimens with varying combinations of bedaquiline (B), levofloxacin (L) or moxifloxacin (M), linezolid (L), clofazimine (fxC), delamanid (D), and pyrazinamide (Z) (BLMZ, BLLfxCZ, and BDLLfxZ) (16, 17).

## 2 Literature identification and selection

As this article was designed as a narrative review, a formal systematic search strategy and predefined study-selection protocol were not applied. Relevant literature was identified through targeted searches of PubMed and MEDLINE, supplemented by screening the reference lists of relevant articles and reports. Literature published up to 2025 was considered, focusing on post-tuberculosis lung disease (PTLD), pulmonary rehabilitation, and key components of comprehensive rehabilitation, including exercise training, breathing and airway-clearance strategies, nutritional support, psychological and psychosocial care, patient education, and tele-rehabilitation. Studies were selected purposively according to their relevance to the scope of the review, with priority given to studies directly evaluating pulmonary rehabilitation or rehabilitation interventions in individuals with post-TB pulmonary impairment. Where PTLD-specific evidence was limited, relevant evidence from established pulmonary rehabilitation practices and other chronic respiratory diseases was included to inform potential application to PTLD. Relevant guidelines, consensus statements, and reports from the World Health Organization and professional respiratory organisations were also considered. No formal risk-of-bias assessment or quantitative evidence synthesis was undertaken.

## 3 Pulmonary rehabilitation for post-tuberculosis lung disease

The full spectrum of economic, psychological, and social complications experienced by TB survivors after treatment remains poorly understood. Many TB survivors still experience long-term symptoms (58%), particularly chronic cough (51%), and disabilities (83.5%), particularly in social participation (65.5%) and mobility (58.5%), even after microbiologic cure (18). These conditions lower their quality of life, limit daily functioning, and lead to social and economic hardship. Pulmonary rehabilitation (PR) emerges as a critical intervention. The primary objectives of PR are to (1) alleviate symptoms, (2) restore functional abilities, and (3) improve overall quality of life (19). Therefore, incorporating PR in long-term TB care can improve activity tolerance, respiratory function, and overall well-being. It can also reduce financial and social stress by helping TB survivors return to work or daily routines.

To date, relatively few studies, including two ongoing clinical trials, have explored pulmonary rehabilitation programs (PRPs) for post-TB lung disease (PTLD) (Table 1). These programs target individuals with symptoms like dyspnea and reduced lung function, aiming to improve health-related quality of life (HRQoL) (20–28). PRPs typically include aerobic exercises (e.g., walking, cycling) and some programs included resistance training and warm-up and cool-down routines. All the program includes educational components on lung health, breathing techniques, and symptom management, among others. Additionally, certain programs offer specialized components such as non-invasive ventilation, inspiratory muscle training (IMT), psychological support, and personalized diet plans (23, 24). Program durations range from 3 to 12 weeks, with 1–5 supervised sessions weekly (20–28). All the programs also encourage additional home-based sessions.

**Table 1:** Pulmonary rehabilitation programs for post-TB patients.

Pulmonary Rehabilitation Program (PRP)	Study design	Results	Reference
Exercise training: **Level walking (15 min)**—90% of initial 6MWT intensity Upper and lower extremities (30 min) **Breathing training:** Relaxation, pursed-lip breathing, and slow-deep breathing **Education (20 min):** Lung anatomy, physiology of various lung impairments, exercise physiology, benefit and methods of daily training, nutrition, drug therapy, oxygen therapy, how to cope with exacerbation, and how to manage daily lives.	**Target:** Post-TB Elderly >70 years old; non-COPD lung disorder vs. COPD **Duration:** 9-week **Frequency:** Once per week, physiotherapist- and respiratory physician-supervised sessions	Non-COPD lung disorder vs. COPD Pulmonary function tests **FEV_1_:** +28 mL vs. +30 mL **FVC:** +72 mL vs. +58 mL Blood gas analysis **Po_2_:** +0.5 mmHg vs. +1.2 mmHg **Pco_2_:** −0.5 mmHg vs. −0.3 mmHg **Exercise capacity** **6MWT:** +42 m vs. +47 m Dypsnea **Transition dypsnea index score:** +1 vs. +1 **Medical Research Council (MRC) Dyspnea Scale:** −0.3 vs. −0.2 Health-related quality of life **Activity of daily living (ADL):** +0.3 vs. +0.4	(20)
Exercise training: **Walking (30 min)**—80% of initial ISWT intensity with progressive increment. **Resistance training for the upper and lower limbs (eight to ten repetitions)**—Sit-to-stand, step-ups, biceps curls, and upright rowing with weights (progressive increment of weights upon achieving three sets). **Education:** Information on PTB and chronic respiratory diseases, causes and effects, self-management, avoiding adverse exposures, exercise, and diet.	**Target:** Post-TB and MRC dyspnea scale ≥ grade 2 **Duration:** 6-week **Frequency:** Home-based PR is conducted five times per week, with patients visiting clinic settings twice weekly for physiotherapist-supervised sessions.	Exercise capacity **Incremental Shuttle Walk Test (ISWT):** +90 m **Sit-to-Stand test:** −2.55 s Health-related quality of life **Clinical COPD Questionnaire (CCQ):** Total score −0.95, symptom score −0.93, mental state score −1.22, functional state score −0.83 **Karnofsky Scale Score:** +13.79% **Patient Health Questionnaire-9 (PHQ-9):** −3.21 Biometrics **Body Mass Index (BMI):** +0.63 kg/m^2^ **Mid-upper arm circumference:** +0.06 cm	(21)
Exercise training with progressive increment (90 min): **Lower limb training**—leg-ergometry and treadmill walking **Upper limb training**—arm-ergometry and free weights. **Simultaneous upper and lower limb training**—semi-recumbent whole body exerciser. **Education:** Breathing exercises, energy conservation, lung health, medications and stress management.	**Target:** Post-TB patients experiencing symptoms of dyspnea, with or without cough, due to chronic lung impairment **Duration:** 8-week **Frequency:** Three days a week, supervised sessions	Pulmonary function tests **FEV_1_, FVC, and FEV_1_/FVC:** No significant changes Exercise capacity **6MWT:** +38 m Health-related quality of life **Chronic respiratory disease questionnaire (CRQ):** +1.75 Dypsnea assessment **MRC:** No significant change	(22)
**Specialist nurse training:** Non-invasive mechanical ventilation and/or oxygen-therapy **Exercise training:** Cycle ergometer (five sessions per week, 30 min each: 5 min warm-up, 20 min training and 5 min warm-down), supervised by respiratory therapist **Education (two sessions):** Lifestyle, physical activity and maintenance programmes. Optional: **Inspiratory muscle conditioning:** Threshold-loading device Breathing exercises Airways clearance **Psychological support** (three sessions per week) **Relaxation techniques** (five sessions per week) **Nutritional counselling** (personalised diet)	**Target:** Post-TB, Impaired respiratory function (COPD and respiratory failure) vs. Normal respiratory function **Duration:** 3-week	Impaired respiratory function vs. Normal respiratory function Pulmonary function tests **FEV_1_:** +100 mL vs. +200 mL **FVC:** +100 mL vs. +300 mL **FEV_1_/FVC:** +1.0 vs. +3.5 Exercise capacity **6MWT:** +35 m vs. 59 m Blood gas analysis **Po_2_:** +4.7 mmHg vs. +18.6 mmHg **Pco_2_:** −1.7 mmHg vs. −5.0 mmHg Dypsnea assessment **Modified Borg dypsnea:** −1 vs −1.5	(23)
Exercise training: **Activities:** Brisk walking and stationary cycling; Strength training with weights for both upper and lower limbs. **Intensity:** Progressive increment **Total Time:** 1 h per session **Frequency:** 5 days a week **Structure:** High-intensity exercises interspersed with lower-intensity periods. **Inspiratory Muscle Training:** **Device:** Philips Respironics threshold inspiratory muscle trainer (IMT) **Intensity:** High-intensity IMT set at 30% of maximum inspiratory pressure with gradually increasing load **Total Time:** 21 min per day **Frequency:** 7 days a week **Structure:** Each cycle consists of 30 repetitions of resistive breathing for 3.5 min, followed by a 1 min rest. This cycle should be repeated twice, three times daily. **Education:** Breath training, stress management, and lung health.	**Target:** Post-TB with breathlessness on exertion (MRC scale) and pulmonary function impairment on spirometry **Duration:** 12-week **Control group:** Own exercises and running without any supervision **Intervention group:** Physiotherapists-supervised PRP	Control group vs. Intervention group Pulmonary function tests: **FEV_1_:** +41 mL vs. +239 mL **FVC:** +159 mL vs. +310 mL **FEV_1_/FVC:** +1.98 vs. +0.89 Exercise capacity: **6MWT:** +58 m vs. +134 m Health-related quality of life: **St George respiratory questionnaire (SGRQ):** −13.01 vs. −12.9	(24)
Exercise training **Activity:** Walking **Intensity:** 80% of initial 6MWT with progressive increment of 5% per week till reach 100%. **Frequency:** 20 min, twice a day **Structure:** Starting speed 11–13 points on the Borg scale for the initial 2 weeks and progressively increased up to 15 on the Borg scale for the final 2 weeks. Resistance training Target muscles: **Lower Body**—Quadriceps, hamstrings, hip flexors, abductors, and extensors **Upper Body**—Deltoid, triceps, and biceps muscles **Intensity**: 50%–70% of the participant’s 1 repetition maximum (1-RM), determined for each exercise. Intensity was progressively increased over the four weeks: ○ 50% of 1-RM in the first week ○ 60% of 1-RM in the middle two weeks ○ 70% of 1-RM in the fourth week **Frequency:** Three sets of 8–10 repetitions for each exercise with rest period of 2–3 min between sets Flexibility training **Stretching exercise:** Seated single-leg hamstring stretch, standing quadriceps stretch, chest stretch, overhead reach stretch, and wall cat stretch **Frequency:** Each stretch 15–30 seconds **Structure:** Five minutes at both the beginning and the end of each exercise session **Education:** Self-management of symptoms, relaxation techniques to minimize dyspnea, cessation of smoking, and nutritional suggestions.	**Target:** Post-TB with shortness of breath on exertion and limitations in the activities of daily living **Duration:** 4-week **Frequency:** The training was done under the supervision of a physical therapist on 4 weekdays. On the weekend, the patients were instructed to perform walking as prescribed during supervised sessions.	Intervention Group vs. Control group (Usual activities) Pulmonary function tests FEV_1_: +150 mL vs. +90 mL FVC: No changes FEV_1_/FVC: No changes Exercise capacity 6MWT: +197 m vs. +8 m Health-related quality of life Short form-36 questionnaire (SF-36) ○ Physical functioning: +46.5 vs. +3.1 ○ Role physical: +29.7 vs. +3.1 ○ Bodily pain: +44.5 vs. +3.3 ○ General health: +39.5 vs. +3.4 ○ Vitality: +26.1 vs. +2.1 ○ Social functioning: +32.9 vs. +2.6 ○ Role emotional: +12.4 vs. +2.5 ○ Mental health: +23.3 vs. +2.9 Dypsnea assessment MRC: −3.36 vs. −3.1	(25)
**Exercise training:** Cycle ergometer (at least 15 sessions), 30 min each: 5 min warm-up, 20 min training and 5 min warm-down) **Education** (at least 3 sessions)**:** Inhalation therapy, maintenance physical exercises, daily physical activities, bronchial hygiene techniques, and home oxygen therapy.	**Target:** Post-TB lung disease **Duration:** 3-month	Intervention Group vs. Control group **Pulmonary function tests** **FEV_1_:** +230 mL vs. −270 mL **FVC:** +240 mL vs. −260 mL **FEV_1_/FVC:** 3.1 vs. −5.2 **TLC:** +190 mL vs. −60 mL **RV:** +2200 mL vs. −970 mL **DLCO:** +3.97 vs. −4.33 Blood gas analysis **Po_2_:** +4 mmHg vs. −2.8 mmHg **SaO_2_:** +1.9% vs. −1.1% Exercise capacity **6MWT:** +58.4 m vs. +4.5 m	(26)
**Randomised Controlled Trials (On-going)**			
Exercise training: **Walking**—85% of initial ISWT with progressive increment **Cycling** Resistance training: **Upper body**—Weights, Theraband **Lower body**—Sit-to-stand, stepping up-down **Education (15 min per session)**—Drug treatment and inhalers, Healthy nutrition; Coping strategies for post-TB lung disease (PTBLD); Chest infections; Pulmonary rehabilitation program; Information about the respiratory system; Information about TB; Living with and after TB; Co-diseases with TB; Role of exercise in PR; Cigarette smoking and biomass smoke exposure	**Target:** Post-TB lung disease; MRC dyspnea scale ≥ grade 2 **Duration:** 6-week **Frequency:** Twice weekly, supervised 2 h sessions, including one strength training session and daily walking at home.	Outcome measures will be performed at baseline, 6 and 12 weeks. **Exercise capacity and muscular strength:** ISWT and five-repetition sit-to-stand test **Symptoms:** Dyspnoea with MRC Dyspnoea Scale and chest pain with Brief Pain Inventory (Short Form) **Health-related quality of life:** Clinical COPD Questionnaire (CCQ), COPD Assessment Test (CAT), and EuroQol 5-Dimension 5-Level questionnaire (EQ5D5L) **Psychological well-being:** Hospital Anxiety and Depression Scale (HADS) **Productivity:** Work Productivity and Activity Impairment (WPAI) questionnaire **Physical activity:** Monitored using an ActiGraph activity tracker for 1 week before the program and at the 6-week follow-up. **Cost/benefit analysis:** Calculated based on the costs to set up, run, and maintain the pulmonary rehabilitation program, with average cost per patient determined.	(27)
Warm-up and cool-down (10–15 min): **Stretching exercise**—Hamstring stretch, Quadriceps stretch **Flexibility exercise**—Marching on the spot; Heel digs, Toe taps, High knee marching, Side bends, Arm stretches, Alternate arm punches, Side taps, Shoulder rolls, Elbow circles Exercise Training: **Two stations:** Stationary cycling (load-adjustable) and ground-based walking (starting intensity 85% of initial ISWT). **Duration:** 10 min per station or until reaching a Borg dyspnoea score of 4–6 (moderate to very severe). **Intensity:** Continuous exercise, with rest or lower intensity as needed. Resistance Training: **Upper limb exercises:** Pull-ups and biceps curls. **Lower limb exercises:** Sit-to-stand and step-up. **Frequency:** Three sets of 8–12 repetitions per exercise. **Education (1 h):** Normal anatomy and physiology of the lungs; Pathophysiology of chronic lung disease; Tuberculosis and how it causes lung damage; Coping with chronic lung disease and coping with stress; Avoidance of risk factors for chronic lung disease; Early recognition and treatment of exacerbations; Strategies for managing breathlessness; Energy conservation during activities of daily living; Role and rationale for medications and devices; Benefit of exercise and physical activities; Healthy food intake; Secretion clearance techniques.	**Target:** Post-TB with chronic respiratory symptoms and radiological evidence of lung parenchymal damage. **Duration:** 6-week **Frequency:** Twice weekly supervised, Participants are encouraged to continue endurance and resistance exercises independently at home.	Outcome measures will be performed at baseline, 6 and 12 weeks. **Health-related quality of life:** COPD Assessment Test (CAT), Clinical COPD Questionnaire (CCQ), and EQ-5D-5L for quality of life. **Psychological well-being:** Hospital Anxiety and Depression Scale (HADS) and Patient Health Questionnaire (PHQ-9) **Productivity:** Work Productivity and Activity Impairment (WPAI) questionnaire **Exercise capacity/physical function:** 5-repetition sit-to-stand test (FTSTS) and Endurance Shuttle Walking Test (ESWT) **Symptoms:** MRC dyspnoea scale **Physical activity:** ActiGraph wGT3X-BT monitors will track participants’ physical activity over 1 week pre- and post-intervention. **Cost/benefit analysis:** Includes calculating single and recurrent costs for PR program setup and maintenance, with average costs calculated separately.	(28)

FEV1, Forced Expiratory Volume in 1 s; FVC, Forced Vital Capacity; TLC, Total Lung Capacity; RV, Residual Volume; DLCO, Diffusing Capacity of the Lungs for Carbon Monoxide; Po_2_, Partial Pressure of Oxygen; Pco_2_, Partial Pressure of Carbon Dioxide; SaO_2_, Arterial Oxygen Saturation; 6MWT, 6 Min Walk Test; ISWT, Incremental Shuttle Walk Test; MRC, Medical Research Council; COPD, Chronic Obstructive Pulmonary Disease.

The American Association of Cardiovascular and Pulmonary Rehabilitation (AACVPR) recommends comprehensive outcome assessment to evaluate the multidimensional effects of PR (29). These outcome measures can be viewed within the framework of the International Classification of Functioning, Disability and Health (ICF), which categorises rehabilitation outcomes into impairments of body function and structure, activity, and participation. Applying the ICF framework provides a more holistic evaluation of rehabilitation by capturing physiological recovery together with functional performance and social reintegration.

Within the impairment domain, pulmonary function is commonly assessed using spirometry, including forced expiratory volume in one second (FEV_1_), forced vital capacity (FVC), and the FEV_1_/FVC ratio, together with blood gas analysis (20–28). The AACVPR also recommends plethysmography for measuring total lung capacity (TLC), residual volume (RV), and functional residual capacity (FRC), as well as diffusion capacity for carbon monoxide (DLCO), although only one recent PTLD rehabilitation programme has incorporated these advanced assessments (26). Symptom burden is commonly evaluated using the Modified Medical Research Council (mMRC) Dyspnoea Scale, Transition Dyspnoea Index (TDI), and Borg Dyspnoea Scale (20–28), while psychological impairment has received comparatively less attention. Only one published programme assessed depression using the Patient Health Questionnaire-9 (PHQ-9) (21), whereas ongoing clinical trials have expanded mental health assessment by incorporating the Hospital Anxiety and Depression Scale (HADS) (27, 28).

Within the activity domain, exercise capacity is most frequently evaluated using the Six-Minute Walk Test (6MWT), Incremental Shuttle Walk Test (ISWT), and Sit-to-Stand Test (20–28). More recent clinical trials have further incorporated objective physical activity monitoring using ActiGraph devices to better quantify daily functional performance (27, 28).

The participation domain reflects the broader impact of rehabilitation on patients' daily lives. HRQoL is commonly assessed using validated questionnaires including the Clinical COPD Questionnaire (CCQ), COPD Assessment Test (CAT), EuroQol-5 Dimension-5 Level (EQ-5D-5L), Short Form-36 (SF-36), and St George's Respiratory Questionnaire (SGRQ) (20–28). Ongoing studies have expanded this domain by evaluating work productivity using the Work Productivity and Activity Impairment (WPAI) questionnaire, together with cost-benefit analyses examining the resources required to establish, operate, and sustain PRPs (27, 28). In addition, these trials assess patient outcomes six weeks after programme completion, providing valuable insight into the durability of rehabilitation benefits beyond the intervention period.

Overall, current evidence demonstrates that PRP benefits a broad spectrum of TB survivors, including patients with COPD, non-COPD chronic respiratory disease, older adults, and even individuals with relatively preserved pulmonary function (20, 23). Compared with usual care or conventional exercise, structured PR consistently improves exercise capacity, pulmonary function, and HRQoL (24–26). However, these findings should be interpreted with caution because several methodological limitations and potential confounding factors are inconsistently reported across published studies. Considerable heterogeneity exists in participant characteristics, disease severity, rehabilitation protocols, programme duration, and outcome measures, limiting direct comparison between studies. Furthermore, information regarding concomitant medications, such as bronchodilators, corticosteroids, or other adjunctive therapies that may influence rehabilitation outcomes, is often lacking. Adherence to supervised and home-based rehabilitation programmes, a key determinant of programme effectiveness, is also seldom evaluated using standardised measures. Although PR is generally considered safe, adverse events related to exercise training are infrequently reported, making comprehensive assessment of programme safety difficult. Participant characteristics are frequently inadequately described, with inconsistent reporting of disease severity, radiological extent of lung damage, bronchiectasis, fibrosis, pulmonary function impairment, and other standardised clinical severity measures. In addition, while current studies primarily focus on impairment and activity outcomes, participation outcomes, including return to work, long-term social participation, sustained functional recovery, and patient-reported outcomes, remain comparatively underrepresented. Future studies should adopt more standardised reporting of participant characteristics, disease severity, prescribed medications, rehabilitation adherence, adverse events, and long-term follow-up while incorporating outcome measures across all ICF domains to strengthen the evidence base and improve comparability of PR programmes for PTLD.

The comprehensive pulmonary rehabilitation programme for post-tuberculosis lung disease is illustrated in Figure 1. Comprehensive PR extends beyond exercise training alone and integrates multiple evidence-based interventions, including exercise training, breathing retraining, nutritional support, psychological care, patient education, and telemedicine, to address the complex physical, functional, psychological, and social consequences of PTLD. By adopting a multidisciplinary, patient-centred approach, comprehensive PR aims to optimise functional recovery, improve health-related quality of life, promote long-term self-management, and facilitate reintegration into daily activities and society. The following sections discuss each component of comprehensive pulmonary rehabilitation in greater detail.

**Figure 1:**
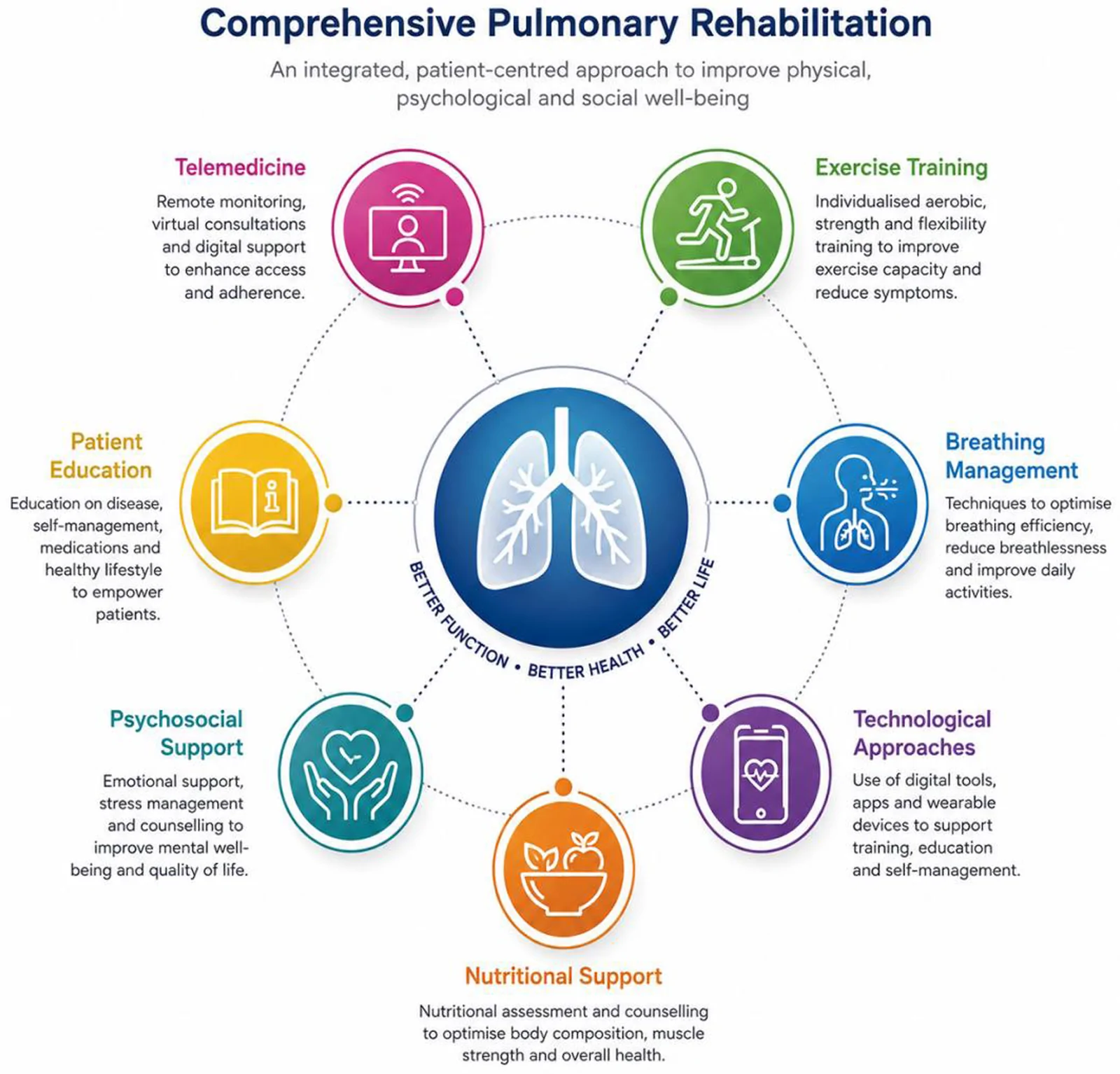
Comprehensive pulmonary rehabilitation.

### 3.1 Exercise training

Exercise training is considered the basis of PR (30). It helps improve cardiovascular and muscular endurance, which is essential for combating the deconditioning that often occurs after prolonged illness. It involves building both strength and stamina through targeted workouts to counteract muscular weakness and enhance coordination, allowing post-TB patients to engage in daily activities with reduced breathlessness (21).

Exercise training includes a combination of warm-up period, aerobic exercises such as walking and cycling, resistance training of upper and lower limbs, and cool-down period. During pre- and post-exercise sessions, 5 to 15 min are dedicated to stretching and flexibility exercises (25, 26, 28). Stretching exercises, such as the seated single-leg hamstring stretch, standing quadriceps stretch, chest stretch, overhead reach stretch, and wall cat stretch, are held for 15–30 s each (25). Flexibility exercises include marching on the spot, heel digs, toe taps, high knee marching, side bends, arm stretches, alternate arm punches, side taps, shoulder rolls, and elbow circles (28).

Walking is the primary activity in aerobic exercises, and walking intensity is crucial to build the endurance (20–25, 27, 28). The starting intensity can be set at 80%–90% of the initial 6MWT or ISWT, with progressively increasing intensity till 100%. It is recommended to start walking at Borg Scale of 11–13 and progressively increased up to 15 (25). The duration of walking sessions ranges from 15 to 30 min. The frequency of walking is based on the setting, where supervised programs typically involve sessions once to four times weekly, while home-based programs may include daily sessions, either once or twice per day (20–25, 27, 28). Some PRP also include stationary cycling using a cycle ergometer, performed for 30 min, five times per week (23, 24, 26–28).

Resistance training for the upper limbs consists of biceps curls, upright rowing, pull-ups, and exercises using free weights or Therabands, targeting the deltoid, triceps, and biceps muscles. For the lower limbs, resistance training includes sit-to-stand, step-ups, leg ergometry, and treadmill walking, focusing on the quadriceps, hamstrings, hip flexors, abductors, and extensors. Most programs recommend performing each exercise in three sets of 8 to 12 repetitions, with 2 to 3 min of rest between sets (20–25, 27, 28). The training intensity is progressive, starting at 50% of the one-repetition maximum (1-RM) and increasing weekly (25).

In general, exercise training should be customized based on their needs, abilities, and goals. To guide the exercise plan and track progress, lung function and exercise endurance should be evaluated both before and after rehabilitation. Training should follow general fitness guidelines, with intensity gradually increasing beyond daily activity levels to build strength and aerobic capacity, while avoiding overexertion (31). Additionally, the success of exercise training relies on the consistent participation and commitment of the patients. Incorporating home-based exercises that require minimal or no equipment, along with culturally and demographically relevant activities such as rhythmic movements (dancing, wushu), ball sports (volleyball, basketball), and recreational activities (running, cycling, gymnastics), can help maintain engagement and promote long-term adherence to the program (32).

### 3.2 Breathing management techniques

The scarring and damage caused by TB, such as pulmonary fibrosis, atelectasis, bronchiectasis, and airway stenosis, can cause irreversible damage to lung structure and result in permanent reduction in lung function. Effective breathing management techniques are essential to help post-TB patients regain control over their breathing, improve oxygen exchange, and reduce the sensation of breathlessness. Many of these breathing techniques were originally developed for treating COPD patients, as COPD-like symptoms are a common complication in post-TB lung disease.

Diaphragmatic breathing, also known as abdominal breathing, focuses on engaging the diaphragm more effectively during inhalation. This technique significantly increases diaphragm movement and reduces uncoordinated respiratory patterns (33). It enhances tidal volume and oxygen saturation, lowers respiratory rate, and improves ventilation efficiency. For patients with reduced lung capacity and increased work of breathing, diaphragmatic breathing improves air intake efficiency (34). By promoting deeper, slower breaths, this technique reduces reliance on accessory muscles (such as those in the neck and chest), conserving energy, and decreasing the burden on the lungs.

Pursed-lips breathing is another effective technique for managing shortness of breath. This method involves inhaling slowly through the nose and exhaling gently through pursed lips (35). It slows exhalation, prevents airway collapse, and is particularly beneficial for patients with damaged or obstructed airways. By prolonging exhalation, pursed-lips breathing reduces air trapping, increases carbon dioxide elimination, and enhances gas exchange, thereby easing breathlessness (36). The combined practice of diaphragmatic breathing and pursed-lips breathing facilitates the elimination of residual air, improves gas exchange, strengthens respiratory muscles, and promotes recovery of lung function in COPD patients (37).

Breathing exercises are often combined with physical activities such as clenching fists, bending and extending elbow joints, and coordinating inhalation with bending movements and exhalation with straightening movements in sitting, standing, or lying down position (38). They effectively enhance muscle strength, improve coordination, increase chest mobility, enhance airway clearance, aid in sputum removal, and boost the strength and endurance of ventilatory muscles (39, 40).

PR incorporates basic stretching exercises to enhance musculoskeletal function, posture, and flexibility. Prolonged illness and inactivity can cause muscle weakness and stiffness in post-TB patients, especially in the upper back, shoulders, and chest. Stretching activities encourage more efficient breathing patterns, improve posture, and ease stress in these areas (30). For example, shoulder and upper body stretches can expand the chest, facilitate deeper breathing, and reduce the effort required for respiration (30). These exercises are easy to perform and adaptable to various settings, making them highly accessible for patients throughout their rehabilitation process.

### 3.3 Advanced technological interventions in pulmonary rehabilitation

Recent advancements in pulmonary rehabilitation have integrated various technological interventions such as inspiratory muscle training (IMT), airway clearance techniques (ACT), non-invasive mechanical ventilation (NIV), and neuromuscular electrical stimulation (NMES) to support patients with chronic respiratory conditions.

IMT is a targeted exercise program that strengthens breathing muscles, including the diaphragm and intercostals to improve endurance and physical performance. IMT includes resistance training (breathing against a set resistance threshold), endurance training (rapid breathing and deep inhalation, also known as normocapnic hyperpnea), and threshold load training (airflow allowed only when a set pressure is reached. These trainings help to strengthen, improve stamina, and increase the force-generating capacity of the inspiratory muscles, respectively. One PRP utilized the Philips Respironics inspiratory muscle trainer, starting at 30% of maximum inspiratory pressure, with gradually increased load. Each session included two cycles of 30 repetitions of resistive breathing for 3.5 min, followed by a 1 min rest, conducted three times daily, seven days a week (24). With consistent training, IMT lowers activity fatigue over time and strengthens respiratory muscles.

ACT include a variety of methods to help clear mucus from the airways. Conventional approaches involve postural drainage, percussion, vibration, coughing, and forced expiratory technique (FET). Modern breathing exercises include the active cycle of breathing technique (ACBT) and autogenic drainage. Instrumental methods, including positive expiratory pressure (PEP), oscillatory PEP, intrapulmonary percussive ventilation (IPV), high-frequency chest wall oscillation (HFCWO), and mechanical insufflation-exsufflation (MI-E), which provide additional support for effective mucus clearance (41, 42). ACT should be emphasized more in patients with post-TB bronchiectasis, as this condition is characterized by reduced lung function and a higher reliance on mucolytics compared to other forms of bronchiectasis (43). A post-TB bronchiectasis patient underwent a PR program consisting of the ACBT, with five 30 min sessions per week for two weeks helped to relieve dyspnea, improve phlegm clearance, and increase physical activity (44).

NIV methods commonly used for patients with COPD include non-invasive positive pressure ventilation (NPPV) and high-flow nasal cannula oxygen therapy (HFNC). NPPV involves connecting a ventilator to provide positive pressure-assisted ventilation through non-invasive interfaces, such as nasal masks, oral-nasal masks, or full-face masks (45). While oxygen supply via face mask with a bag is routinely used in these patients, it may be inadequate for some, particularly those who require higher inspiratory flow. HFNC is a newer technology that uses an air-oxygen blender to deliver a high flow of heated and humidified oxygen through a nasal cannula. This method can improve oxygenation and reduce the need for intubation and invasive mechanical ventilation in certain cases (46). In 2008, the new consensus on lung rehabilitation in Argentina recommended NIV for patients with COPD or poor exercise tolerance. This approach can alleviate breathing difficulties, reduce the workload of respiratory muscles, improve exercise endurance, and enable higher exercise intensity (36).

NMES is a modality that delivers electrical impulses to the nerves of the thigh muscles, causing them to contract in a manner that mimics the natural action potentials generated by the central nervous system. NMES is generally safe and well tolerated by most patients. It has been used mainly for strengthening and maintaining muscle strength and prevent neuromyopathy, a condition in which muscle strength deteriorates due to long-term illness or immobilization. NMES typically recruits fast-twitch muscle fibers first, which are responsible for quick, powerful movements, unlike voluntary exercise that initially activates slow-twitch fibers during endurance activities (47). NMES has been shown to improve physical function and alleviate breathlessness in COPD patients (48). Additionally, NMES devices are small, portable, and relatively inexpensive, making them a feasible option for home-based rehabilitation and long-term management.

### 3.4 Nutritional support

Malnutrition is a significant factor contributing to TB cases worldwide, with over two million TB cases linked to undernutrition (49). Malnutrition makes individuals more susceptible to developing and progressing TB and reduces their ability to respond to treatment, while TB further worsens their nutritional status (50). In TB patients, malnutrition can be categorized as hunger-related or disease-related (51). Hunger-related malnutrition stems from food insecurity, leading to deficiencies in both macronutrients and micronutrients that impair innate and adaptive immune responses to Mtb infection (52–54). Disease-related malnutrition, resulting from infection-induced weight loss, catabolism, cachexia, loss of appetite, and malabsorption, can significantly impact the effectiveness of anti-TB drugs. Malnutrition lowers protein levels, particularly albumin, reducing drug binding and leading to faster clearance, which decreases blood drug concentrations. Additionally, impaired intestinal absorption and liver metabolism further contribute to inadequate drug exposure, ultimately reducing treatment efficacy. Conversely, if higher doses are administered to compensate, there is a risk of overdose due to altered drug distribution and metabolism (51, 55). Undernutrition increases the risk of unsuccessful TB treatment (56), and higher rates of treatment failure and mortality have been observed in malnourished patients with MDR-TB (57).

The WHO introduced nutritional guidelines for TB patients in 2013, recommending periodic nutritional assessments and counseling during treatment. These assessments are crucial for nutrition management and are typically standardized to global norms (58). Nutritional assessment components include anthropometric indicators [e.g., body mass index (BMI), mid-upper arm circumference, skin-fold thickness, and presence of edema], evaluation of physical and cognitive functions, dietary intake, and nutritional status [e.g., Mini Nutritional Assessment (MNA) and the Subjective Global Assessment (SGA)] in TB patients (59).

BMI is commonly used to assess nutritional status due to its ease of use and low cost, compared to more complex methods like dual-energy x-ray absorptiometry (DXA) and bioelectrical impedance analysis (BIA) (60). Some studies indicate a strong association between BMI and TB risk. Underweight individuals (BMI <18.5 kg/m^2^) had a significantly higher risk of developing TB compared to those of normal weight (61), while each unit increase in BMI (within 18.5–30 kg/m^2^) was linked to a decrease in TB incidence (60). Nevertheless, using BMI alone is insufficient for evaluating body composition and some metabolic conditions. BMI cannot distinguish between fat mass and muscle mass or detect changes in body composition, so it may not accurately identify malnutrition in overweight and obese individuals. Some TB patients with higher BMI, including those who are overweight or obese, may still be malnourished, showing reduced fat-free mass, body fat, and leptin levels (51, 62, 63). Cegielski et al. found that low subcutaneous fat and lean muscle mass were associated with a higher TB risk, suggesting that poor nutritional status rather than high BMI alone increases vulnerability (61). Lu et al. found that BMI and prognostic nutritional index (PNI) were found to be protective factors for severe PTB disease (64).

Recently, the Global Leadership Initiative on Malnutrition (GLIM), which combines both phenotypic (e.g., weight loss, muscle mass) and etiological (e.g., inflammation, disease-related factors) criteria, has been a recommended tool for diagnosing malnutrition (65). Additionally, nutritional intervention is recommended in combination with exercise training to enhance rehabilitation, as outlined in the European Society for Clinical Nutrition and Metabolism (ESPEN) guidelines (66). To date, no study has specifically focused on the benefits of nutritional intervention for post-TB patients. However, some studies have demonstrated the benefits of integrating individual dietary counselling in PRP (20–25, 27, 28).

### 3.5 Psychological support

TB is not only a physical health challenge but also poses significant mental health risks. Also, people with mental disorders are more susceptible to TB (67). Approximately 40% to 70% of patients with TB experience clinical anxiety and/or depression following their diagnosis. These psychological symptoms may include irritability, restlessness, low mood, pessimism, and fears of transmitting the disease to family members or facing treatment failure. Due to the financial impact of their treatment on their family, many patients often express feelings of guilt and believe they are a burden. Self-blame and self-guilt are common, and in severe cases, these emotional struggles can lead to distorted self-perceptions or even suicidal ideation and behaviors (68, 69). TB patients with comorbid mental disorders are more likely to exhibit poor medication adherence and delayed treatment, which is linked to acquired drug resistance, the transmission of TB, disease recurrence, and TB-related mortality (70). This issue is especially pronounced in patients with MDR-TB due to the chronic nature of the illness and the side effects of prolonged treatment (71).

Furthermore, patients initiating anti-TB treatment may experience sleep disturbances, including difficulty falling asleep, early awakening, or insomnia, due to the euphoric effects of the medication (72). The persistence of long-term sleep disorders further affects the treatment and recovery of the disease, thereby creating a detrimental cycle. The use of anti-TB drugs may also induce psychiatric symptoms, including hallucinations and delusions (73). These symptoms may include transient and fragmentary hallucinations, feelings of victimization, auditory hallucinations (such as hearing voices), visual hallucinations (perceiving non-existent people or objects), and a persistent sense of being unsafe, stalked, watched, or persecuted.

Stigma in TB refers to the negative attitude or belief directed toward individuals affected by the disease, leading to discrimination and social exclusion. It manifests through blame, shaming, gossip, and rejection, often resulting in isolation. Patients may experience perceived stigma (awareness of societal judgment) and self-stigma (internalized shame), both of which hinder social interaction and treatment-seeking. It can contribute to mental health issues such as anxiety, depression, stress, anger, and, in extreme cases, neuropsychiatric disorders (e.g., impaired consciousness, lethargy, delirium, or comatose state), behavioral abnormalities (e.g., purposeless limb movements, incomprehensible self-talk, moaning, or shouting), acts of violence (e.g., throwing tantrums, fighting, or property vandalism), alcoholism, suicide, and even death. The fear of stigma often discourages individuals from getting tested, disclosing their TB status, or seeking treatment, which in turn leads to further spread of the disease (74).

For patients presenting with psychiatric symptoms, consultation with a psychiatric specialist is recommended. Depending on the severity of the symptoms, sedative-hypnotics may be used to improve sleep, anxiolytic antidepressants to alleviate mood disturbances, or atypical antipsychotics to manage hallucinations and delusions. Treatment should primarily target the underlying condition, with careful monitoring for potential drug interactions to minimize adverse effects.

No direct studies have specifically examined the benefits of psychological support in post-TB patients. Among PRPs, only one has recommended psychological support with three sessions per week, though this remains optional (23). Despite this, most PRPs include at least one educational session addressing stress management or coping strategies (20–28). In COPD patients, interventions such as cognitive-behavioral therapy (CBT), mindfulness, psychoeducation, and mind-body approaches have shown at least partial evidence of efficacy in addressing mental health issues (75).

### 3.6 Patient education/ self-management

Health education is essential for TB patients, as healthcare professionals, including doctors, nurses, nutritionists, therapists, and social workers, provide patients and their families with the knowledge and skills to manage the disease effectively. The education component provides important knowledge about both the structure and function of healthy lungs, as well as pathological impairments, including pulmonary TB and chronic respiratory diseases (CRD), which they are at higher risk of developing due to lung damage following the infection. Understanding the causes and consequences of CRD enables patients to better manage their condition, recognize symptoms early, cope with exacerbations, and seek timely medical intervention (20–28).

Educating post-TB patients on the benefits and methods of daily training is essential for their rehabilitation. Lifestyle modifications, including smoking cessation and reducing alcohol consumption, play a key role in protecting lung health and preventing further damage. Exercise physiology is also important, as regular physical activity improves lung capacity, enhances circulation, and supports respiratory function. Patients should also receive training on maintenance programs, which cover breathing techniques to manage shortness of breath, energy conservation strategies to maintain stamina throughout daily activities, and lung health preservation. Furthermore, teaching patients self-managing their sputum clearance can aid in reducing the frequency of exacerbations and limits unnecessary antibiotic use. Additionally, education on medication management, proper nutrition, and stress reduction techniques helps patients sustain their rehabilitation progress (20–28). This education empowers them to take an active role in their recovery, make informed decisions, and adhere to PRP that are essential for improving their quality of life. It also ensures long-term commitment to PRP and reduces the risk of disease progression, contributing to better overall health outcomes (20–28).

## 4 Telemedicine in pulmonary rehabilitation

Despite the well-established benefits of PR, many patients with chronic respiratory diseases are either not referred to or lack access to PRP. This is primarily due to geographic distance and transportation challenges (i.e., bad roads and high cost), as most accredited PR facilities are located in urban areas, making it difficult for patients in rural or remote regions to participate (76, 77). Furthermore, the lengthy duration and complexity of TB treatment pose significant barriers to patient adherence. Telemedicine offers a solution by enabling patients to access PR programs without the need for long-distance travel, which is especially benefit for those with limited mobility or transportation barriers. Telemedicine support various PR applications, including remote exercise training, telemonitoring, teleconsultations, tele-education, and psychosocial support (78). Telerehabilitation can be conducted through videoconferencing, allowing participants and healthcare providers to see and speak with each other, telephone calls for voice communication, and website-based platforms for accessing information independently. It can be used for long-term monitoring in both primary and maintenance phases of PR, extending up to 12 months. Participants are more likely to complete a telerehabilitation program compared to in-person rehabilitation. Additionally, there appears to be little or no difference between the two methods in improving exercise capacity, dyspnea, and quality of life (79). Moreover, telemedicine ensures adherence to medication schedules, lowers the recurrence rate, hinders the development of DR-TB strains, and decreases patient mortality (80, 81). Telemedicine has made a substantial contribution to the prevention of MDR-TB in Nigeria, thereby reducing disease transmission, enhancing access to medical services, improving medical quality, boosting patient adherence with treatment, and cutting medical costs (82).

Remote exercise training, such as home-based programs monitored via videoconferencing, offers a flexible approach to PR. The Rehabilitation Exercise At Home (REAcH) study demonstrates how telemedicine enables physiotherapists to deliver supervised group-based home exercise training, which includes aerobic exercises and resistance training using simple equipment (e.g., sit-to-stand exercises or lifting bags of rice for upper limb weights) (83). To further enhance home-based rehabilitation, patients can also access exercise videos and educational materials via tablets or interactive web-based platforms and receive asynchronous feedback from healthcare providers (84). Additionally, automated reminders through text messages or mobile app notifications can be sent to patients based on their activity data, prompting them to stay active or complete specific exercises.

Telemonitoring utilizes various technologies to remotely track vital signs, including oxygen saturation, respiratory rate, and symptoms. Motion sensors primarily consist of pedometers and accelerometers, serving to detect bodily movements and objectively quantify physical activity over a certain period of time, thereby tracking the rehabilitation progress (85). A pedometer is a small, affordable device used to track step counts. However, it has limitations, as it only provides basic data like step counts and estimated distances. It does not capture details about physical activity patterns, time spent on activities, or activity intensity. Additionally, pedometers are less sensitive to detecting walking compared to accelerometers (85). Accelerometers, on the other hand, are more advanced and can measure both the quantity and intensity of movement, providing better monitoring of physical activity cycles. These devices are particularly suitable for patients with chronic lung disease, who often experience reduced mobility and require long-term or lifelong exercise training (86).

For example, in a study by Santos et al. (87), physical activity in daily life (PADL) was measured in patients with chronic respiratory conditions, including TB sequelae, through a 4-day telemonitoring protocol using SMARTREAB technology. Patients were equipped with an Android smartphone integrated with an accelerometer and a portable oximeter, both connected via Bluetooth. This setup allowed for the continuous assessment of heart rate, transcutaneous oxygen saturation, and activity-related energy expenditure, measured in metabolic equivalents of task (MET) (87). The use of physical activity monitors, equipped with wearable motion-sensing technology, provide data on step count, activity levels, energy expenditure, and duration of physical activity at various intensities, using a range of thresholds (79). However, while these devices offer quantitative data, their accuracy can be unreliable, especially for shorter measurement periods. Typically, activity must be recorded for at least three days to appropriately characterize the activity patterns of an individual. When thinking about the use of these devices, convenience, patient acceptability, validity, reliability, responsiveness, and cost should all be balanced against the intended goal.

With the advancement of modern technology, wearable devices are utilized to gather data on vital signs, motion assessment, and surrounding environmental parameters of the human body. This enables the monitoring and recording of real-time information regarding patients’ physiological conditions and exercise activities, which proves beneficial for medical prediction, anomaly detection, and diagnosis. This data is stored online and integrated with artificial intelligence algorithms to help analyze patient information, providing decision support for healthcare providers. This allows them to review patient progress, ensure safety and adherence, and adjust rehabilitation strategies as needed. Additionally, insights gained from this data can be incorporated into teleconsultations, enabling healthcare providers discuss progress and customize treatment plans during virtual appointments. Furthermore, regular video or phone consultations enable the healthcare providers to address patients’ challenges or concerns during treatment, ultimately enhancing outcomes (82).

Tele-education in PR plays a crucial role in providing comprehensive educational sessions that cover disease education, lifestyle modifications, self-management techniques, exercise training, breathing retraining, airway clearance techniques, energy conservation strategies, medication advice, dietary recommendations, and psychosocial issues (88). The content can be effectively delivered through user-friendly mobile applications and online resources that incorporate various formats, including videos, interactive modules, text-based information, and online webinars (82). Although specific information about post-TB PR is limited, several voluntary organizations provide valuable resources on PR for chronic respiratory diseases such as American Association of Cardiovascular and Pulmonary Rehabilitation (AACVPR) (https://www.aacvpr.org/Pulmonary-Patient-Resources), American Lung Association (https://www.lung.org/lung-health-diseases/lung-procedures-and-tests/pulmonary-rehab), Lung Foundation Australia (https://pulmonaryrehab.com.au/patient-education/education-topics/), and Asthma + Lung UK (https://www.asthmaandlung.org.uk/living-with/keeping-active/pulmonary-rehabilitation). These resources allow patients to engage with the information at their convenience, enabling them to learn at their own pace and revisit materials as needed.

Moreover, telemedicine-based PR often includes virtual support groups, counseling, and motivational interviewing sessions to help patients cope with emotional and psychological challenges. For example, TB Alert (https://www.tbalert.org/) offers the TB Action Group (TBAG), which connects TB survivors who provide peer support through telephone, face-to-face interactions, or email (https://www.tbalert.org/what-we-do/uk/tb-action-group/). This initiative enables patients to connect with others facing similar experiences, fostering a sense of community, motivation, and shared learning. Such peer support is invaluable in combating the stigma often associated with TB, helping patients feel less isolated and more understood. Additionally, TB Alert provides psychosocial support services, including counseling and mental health resources that equip patients with tools to manage anxiety, depression, and other emotional challenges. Furthermore, behavioral interventions can also be delivered remotely. In 2018, a randomized controlled trial called the ProLife program was launched, which combined motivational interviewing counseling and short message service (SMS) text messaging delivered by health workers. This program aimed to address harmful lifestyle behaviors such as tobacco smoking, alcohol use, and poor medication adherence among pulmonary TB patients (89).

In PR, collaborative care models are vital for optimizing patient outcomes by utilizing a multidisciplinary approach. This model brings together various healthcare professionals, including pulmonologists, respiratory therapists, physical therapists, dietitians, and mental health specialists, each contributing their expertise to develop a comprehensive, personalized care plan. Importantly, telemedicine allows these professionals to collaborate seamlessly, regardless of their physical locations. Additionally, this model supports capacity building and knowledge transfer, especially in low-resource settings. Through telemedicine, specialists can provide remote training and educational sessions, equipping healthcare providers with the necessary knowledge and skills for effective PR management. Online courses and webinars on PR are available globally, such as the certification program offered by the AACVPR (https://www.aacvpr.org/Pulmonary-Rehabilitation-Certificate). These platforms enable healthcare providers to access training materials, expert presentations, and interactive sessions. Telemedicine further facilitates the dissemination of up-to-date information, evidence-based guidelines, and best practices in PR management, ensuring healthcare providers stay informed about the latest advancements in the field.

Telemedicine-based PR offers more flexible, personalized, and accessible care, reducing the burden on both healthcare systems and patients. Since 2017, the World Health Organization (WHO) recommended the use of digital technologies using short message service (SMS), video supported treatment (VOT), and event monitoring device for medication support (EMM) in supporting TB treatment and adherence (90). However, despite its advantages, several challenges remain, particularly in low- and middle-income countries (LMICs) where TB is most prevalent. In these resource-limited settings, access to reliable internet and technology, such as smartphones or computers, is often limited. Many patients lack the necessary devices to participate in telemedicine programs, which can hinder the effectiveness of remote care. Furthermore, digital literacy remains a significant barrier. Many patients may not be comfortable using telemedicine platforms or may lack the skills to navigate online tools and monitoring devices. Safety concerns also arise with telemedicine-based PR, particularly when patients perform exercises independently without the physical presence of a therapist. Issues such as sudden drops in blood pressure, loss of balance, shortness of breath, or dizziness may go unnoticed without direct supervision, potentially leading to injury. Additionally, patients might struggle to properly position or operate wearable devices, which could result in inaccurate data collection and incomplete monitoring. Furthermore, concerns about data privacy and maintaining the quality of care comparable to in-person rehabilitation must be addressed. Lastly, the lack of personal interaction between healthcare providers and patients can diminish the sense of connection and support that in-person therapy provides, potentially impacting patient motivation and the overall effectiveness of rehabilitation programs (76).

## 5 Implementing pulmonary rehabilitation in low-resource settings

Although PR has demonstrated substantial benefits in improving exercise capacity, respiratory symptoms, and health-related quality of life among patients with PTLD, translating these benefits into routine clinical practice remains challenging, particularly in LMICs, where the burden of PTLD is greatest. Conventional multidisciplinary PR programmes developed in high-income countries typically rely on specialised respiratory physicians, physiotherapists, psychologists, dietitians, dedicated rehabilitation facilities, and advanced cardiopulmonary assessment. Such resources are often unavailable or inaccessible in many TB-endemic settings. Therefore, rather than attempting to replicate comprehensive hospital-based programmes, PR should be adapted according to local healthcare capacity while preserving its core evidence-based principles (91, 92).

A pragmatic approach to PR implementation should distinguish between essential and resource-dependent components. Based on the available evidence, the essential components that should be universally available include aerobic exercise training, resistance exercise, breathing retraining, patient education, nutritional assessment and counselling, smoking cessation support, psychosocial assessment, and routine monitoring of symptoms and functional capacity (91, 92). These interventions represent the minimum package of care that can be delivered across different healthcare settings and are likely to provide the greatest functional benefit for most patients. In contrast, interventions such as inspiratory muscle training, non-invasive ventilation, neuromuscular electrical stimulation, advanced pulmonary function testing (e.g., diffusion capacity and plethysmography), specialist psychological services, and respiratory physician-led multidisciplinary clinics may be incorporated where expertise, equipment, and financial resources permit. Such a tiered approach allows healthcare systems to establish evidence-based PR services without delaying implementation while progressively expanding programme capacity as resources become available.

The delivery model should be adapted according to patient needs, disease complexity, patient preference, digital literacy, and local healthcare infrastructure. Centre-based PR remains important for patients with severe functional impairment, complex comorbidities, or those requiring close supervision by a multidisciplinary team. However, reliance on specialised rehabilitation centres may limit accessibility in many LMICs, where services are often concentrated in tertiary hospitals. Alternative models, including community-based, primary care-based, home-based, and telerehabilitation approaches, may improve accessibility and flexibility, and available evidence suggests that several non-centre-based models can achieve outcomes comparable to centre-based PR when core training principles are maintained. Hybrid PR may be particularly useful because it can complement rather than replace centre-based care, for example by adding home-based sessions, replacing selected in-person sessions, providing an alternative when centre attendance is not feasible, or supporting maintenance after a supervised programme. However, hybrid or digital PR requires careful consideration of patient preference, digital literacy, staff workload, cost-effectiveness, and the need to maintain adequate exercise intensity. Therefore, rather than adopting a single delivery model, PR programmes should be personalised according to disease severity, functional status, available workforce, local resources, and the degree of multidisciplinary care required (93).

Successful implementation also depends on strengthening healthcare systems rather than relying solely on specialist services. Workforce shortages remain one of the major barriers to PR delivery in many high TB-burden countries. Consequently, task-sharing approaches should be encouraged, whereby appropriately trained primary care physicians, nurses, community health workers, rehabilitation assistants, and caregivers deliver selected rehabilitation components under specialist guidance when necessary. Integrating PR into existing TB programmes and primary healthcare services may further improve long-term continuity of care without establishing separate rehabilitation infrastructure. Following completion of anti-tuberculosis treatment, patients should undergo routine screening for persistent respiratory symptoms, exercise limitation, nutritional status, and psychological well-being. Those identified with PTLD can then be referred to the most appropriate rehabilitation pathway according to disease severity and available local resources. Such integration may facilitate earlier identification of rehabilitation needs, improve referral pathways, reduce fragmentation of care, and enhance programme sustainability.

Importantly, limited resources should not prevent the implementation of PR. Many effective rehabilitation activities require little or no specialised equipment. Walking programmes, stair climbing, sit-to-stand exercises, step-up training, resistance exercises using elastic resistance bands or readily available household items such as water bottles or bags of rice, together with breathing exercises, can produce clinically meaningful improvements in functional capacity at minimal cost. Exercise intensity may be prescribed using simple measures such as the Borg Rating of Perceived Exertion, heart rate, or symptom-guided progression instead of sophisticated cardiopulmonary exercise testing. Likewise, programme monitoring may rely on practical outcome measures including the 6 Min Walk Test, One-Minute Sit-to-Stand Test, Medical Research Council Dyspnoea Scale, COPD Assessment Test, pulse oximetry, body mass index, and patient-reported quality-of-life questionnaires, all of which are feasible in primary care and district hospital settings (20–28).

Tele-rehabilitation represents another opportunity to improve access to PR. However, its implementation should be adapted according to local digital capacity. Although videoconferencing platforms, wearable activity monitors, and smartphone applications have shown encouraging results, these technologies may be impractical in many LMICs because of limited internet connectivity, inadequate digital infrastructure, financial constraints, and low digital literacy. Consequently, pragmatic alternatives should also be considered. Telephone-based follow-up remains one of the most widely accessible approaches and can be used for symptom monitoring, exercise reinforcement, and patient counselling. Short message service (SMS) reminders may improve adherence to prescribed exercise programmes and follow-up appointments, while pre-recorded exercise videos distributed through smartphones, USB devices, or local health facilities can provide visual guidance without requiring continuous internet access. Where internet connectivity permits, messaging platforms such as WhatsApp may facilitate communication, education, and remote supervision, although their feasibility should be considered in relation to local connectivity and smartphone availability. Caregiver-assisted monitoring, paper-based exercise diaries, and periodic face-to-face reviews may further complement remote follow-up, allowing hybrid rehabilitation programmes to maximise accessibility while maintaining patient safety and programme adherence.

Ultimately, successful implementation of PR requires a structured and context-specific approach that extends beyond the clinical intervention itself. As illustrated in Figure 2, programme implementation should begin by identifying the core evidence-based components of PR that can be delivered within the local healthcare context, followed by an assessment of available resources, workforce capacity, infrastructure, referral pathways, and potential barriers. The intervention should then be adapted to align with local healthcare systems through appropriate task-sharing, utilisation of locally available equipment, and selection of the most feasible delivery model, whether centre-, community-, home-, or hybrid-based. Programme implementation should be accompanied by continuous monitoring of implementation fidelity, patient adherence, clinical outcomes, patient satisfaction, and cost-effectiveness to guide quality improvement and future scale-up. Rather than replicating resource-intensive models developed in high-income countries, healthcare systems should adopt flexible, context-specific rehabilitation programmes that preserve the core principles of multidisciplinary care while adapting delivery methods to local realities. This pragmatic framework provides clinicians, programme developers, and policymakers with practical guidance for establishing sustainable and scalable pulmonary rehabilitation services in settings where the burden of PTLD is greatest.

**Figure 2:**
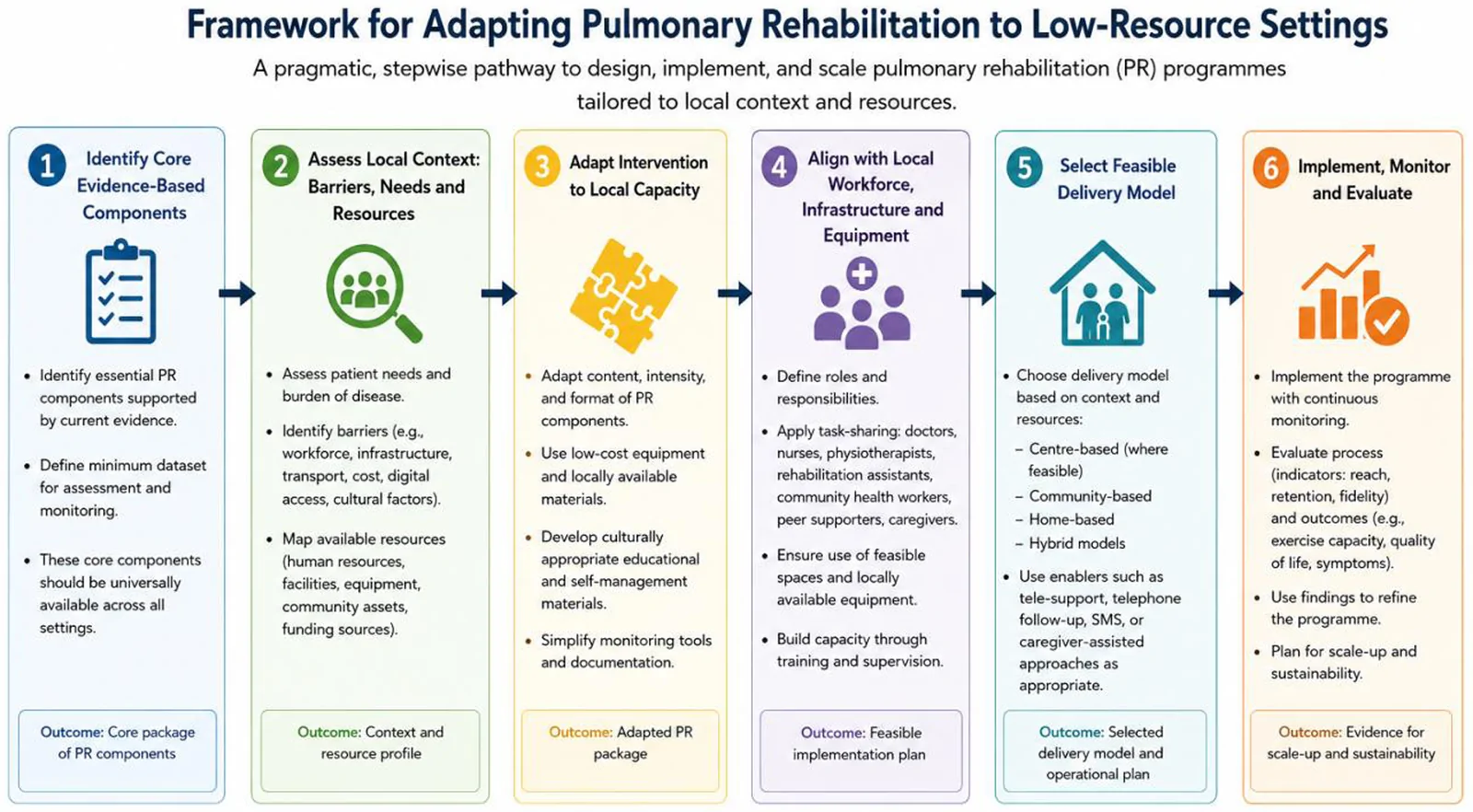
Framework for adapting pulmonary rehabilitation to low-resource settings.

## 4 Conclusion

Patients recovering from TB frequently experience persistent respiratory impairment, reduced exercise capacity, respiratory muscle weakness, and physical deconditioning that continue long after microbiological cure. PR therefore represents a key component of comprehensive post-TB care by improving functional capacity, respiratory symptoms, and health-related quality of life through a multidisciplinary approach incorporating exercise training, breathing retraining, nutritional support, patient education, psychosocial care, and, where appropriate, tele-rehabilitation. However, the greatest burden of PTLD occurs in LMICs, where conventional multidisciplinary rehabilitation programmes are often unavailable. Rather than replicating resource-intensive models developed in high-income settings, PR should be adapted according to local healthcare capacity while preserving its essential evidence-based components. Integrating PR into existing tuberculosis and primary healthcare services, adopting community-, home-, or hybrid-based delivery models, implementing task-sharing approaches, and utilising low-cost exercise strategies and pragmatic tele-rehabilitation can substantially improve programme accessibility, scalability, and sustainability. Future research should focus not only on evaluating the clinical effectiveness of PR but also on implementation strategies, cost-effectiveness, and long-term outcomes to facilitate wider adoption across diverse healthcare settings. Such context-specific implementation will be essential to ensure equitable access to pulmonary rehabilitation and improve the long-term health and quality of life of TB survivors worldwide.
